# Simultaneous determination of bromoxynil and MCPA in commercial samples and raw materials using reversed phase high performance liquid chromatography

**DOI:** 10.1186/s13065-024-01154-x

**Published:** 2024-03-16

**Authors:** Ahmed Yar, Tariq Mahmood Ansari, Faariah Rehman, Asad Raza, Umair Riaz, Rashid Iqbal, Amal M. Al-Mohaimeed, Wedad A. Al-onazi, Muhammad Rizwan

**Affiliations:** 1 Institute of Chemical Sciences, Bahauddin Zakariya University, Multan, 68000 Pakistan; 2 Department of Chemistry, Govt. Post Graduate College Civil Lines, Multan, Punjab 68000 Pakistan; 3 Department of Soil & Environmental Sciences, MNS-University of Agriculture, Multan, 66000 Pakistan; 4 Department of Agronomy, The Islamia University of Bahawalpur, Bahwalpur, 63100 Pakistan; 5 Department of Chemistry, College of Science, King Saud University, P.O. Box 22452, 11495 Riyadh, Saudi Arabia; 6 Institute of Crop Science and Resource Conservation (INRES), University of Bonn, 53115 Bonn, Germany

**Keywords:** Bromoxynil, MCPA, RP-HPLC, Commercial samples, Determination, Validation

## Abstract

This study encompasses a quick, efficient, repeatable and reproducible analytical method for simultaneous determination of Bromoxynil (3, 5-Dibromo-4-hydroxybenzonitrile) and MCPA (2-methyl-4-chlorophenoxyacetic acid) using RP-HPLC with UV-Detector. Bromoxynil + MCPA is one of the most selective post emergent herbicide formulations for the control of important broad leaf weeds infesting small grains (wheat, barley, oats, rye), conservation reserve program areas and grass grown for seed. Optimum weed control is achieved when Bromoxynil + MCPA is applied to actively growing weed seedlings. So, a simple, repeatable, reproducible and efficient simultaneous analytical method was developed for Bromoxynil + MCPA. The developed method was applied for the detection and quantitation of these pesticides in formulations and raw materials with excellent recoveries. It was validated according to ICH Guidelines with excellent linearity R^2^ = 0.992 for Bromoxynil and 0.998 for MCPA. For Bromoxynil, LOD = 1.57 mg/L and LOQ = 5.22 mg/L while for MCPA the LOD = 1.08 mg/L and LOQ = 3.62 mg/L was found. The proposed method has shown high precision (RSD %) 0.06% and 0.11% for Bromoxynil and MCPA respectively while the trueness has been calculated in terms of recovery percentage obtained as “mean value of Bromoxynil 99.53% and MCPA 100.10%” which is excellent under optimized conditions. For repeatability and reproducibility, five replicate readings of standard and sample were taken and had found within acceptable limits of relative standard deviation (RSD ≤  ± 2%). Finally, the robustness of the developed method was determined by changing flow rate and mobile phase ratios that has found within the permissible limits (% RSD NMT 1.5). So, the proposed analytical method has found to be more precise, valid and accurate at commercial scale.

**Graphical Abstract:**

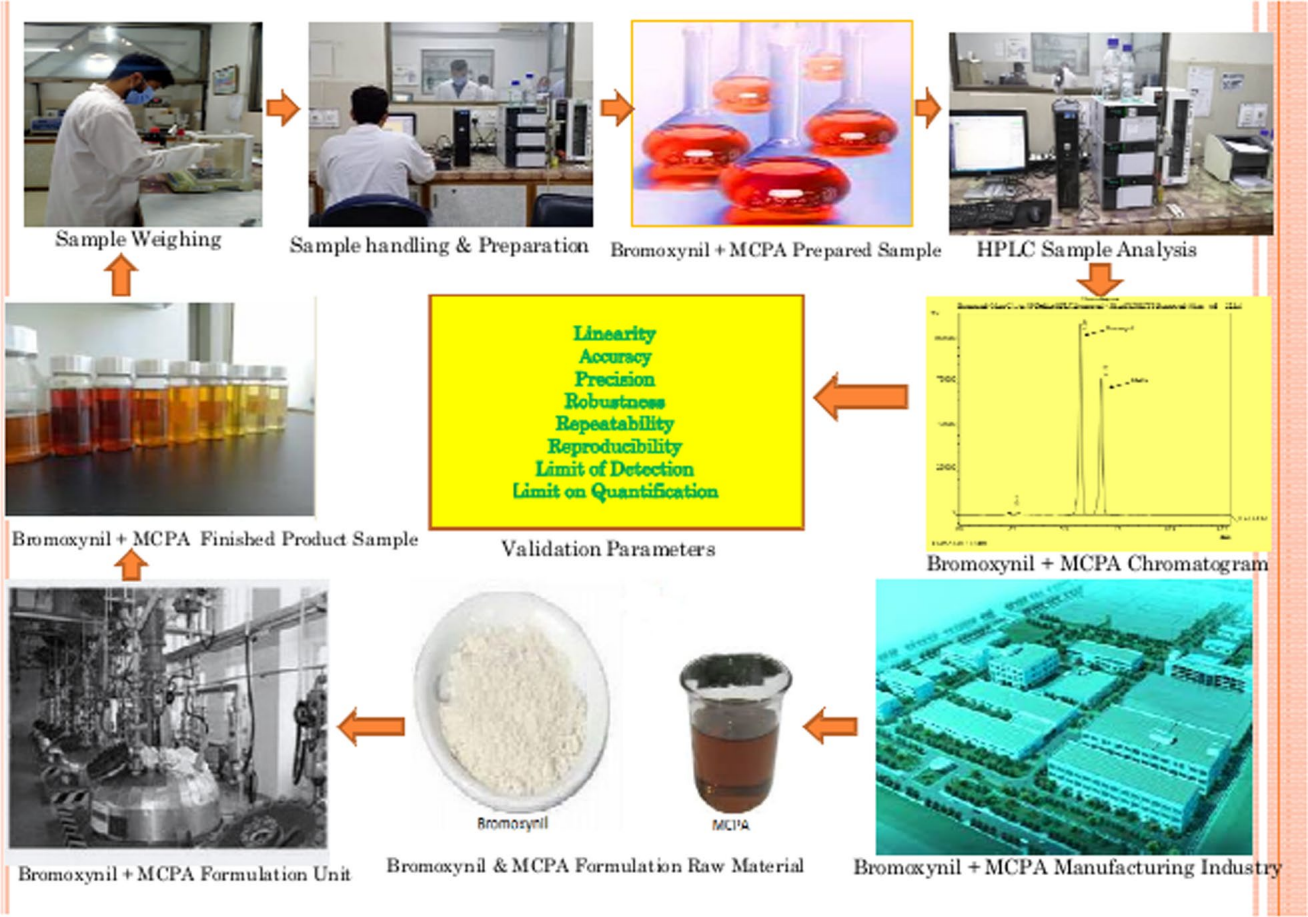

## Introduction

Though pesticides are considered highly toxic chemicals, yet the use of pesticides is not limited to agriculture. These are also being widely employed to control the domestic pests, the disease insect vectors and home gardening. Pesticides can be classified according to their modes of action e.g. organochlorine, organophosphate and carbamates etc. and largely classified into herbicides, insecticides, fungicides, bactericides, avicides and viricides etc. Due to an elevated toxic characteristic, these compounds having health risks on human health and environment. Pesticides negatively affect agricultural workers and trigger social conflicts when used extensively. Primarily, the agriculture workers meet direct and indirect exposure with these chemicals. Common man also comes in contact with these pesticide chemicals by skin which is due to leaking and drifting of pesticides during the mixing and causes very serious threats to the human health [1].

All pesticides have the potential hazard to be harmful to humans, animals, other living organisms and the environment [2, 3]. A book published in 1962, ‘Silent Spring’ portrayed this argument that pesticides have deadly effects the ecosystem. The report was significantly analyzed and it was found that the danger of pesticides is more than actual which guides researcher to find out the way of cropping with minimum use of the pesticides [4]. The labor working in pesticide manufacturing industries, in fields, assassinating of household pests and green house are most commonly affected due to pesticide exposure. At manufacturing site, probability of risk is high as they deal with several dangerous chemicals [5–8]. Different types of health problems arise due to acute poisons and exposure of pesticides [9–12]. Various types of diseases including cancer, diabetes mellitus, respiratory disorders, neurological disorders, reproductive syndromes caused by exposure to pesticides [13–16]. Oxidative stress and chronic disorders are caused due to direct exposure, handling of the pesticides or pesticide residues present in food stuffs [17–20]. The most common exposure scenarios for the pesticide-poisoning cases are accidental or suicidal poisonings and the general community who are exposed through environmental contamination [21]. Insecticides act primarily by disrupting nervous system function, while herbicides target mainly photosynthesis pathways. Over half of herbicides in current use act primarily on light reaction of photosynthesis. Many groups of herbicides act by inhibiting the Hill reaction of electron transport including cyclic urease, triazines, phenylcar-bamates and triazinones [22].

Among the commonly employed pesticides, bromoxynil is considered a highly toxic one due to its ability to accumulate in fatty tissues. As the part of the Prairie Ecosystem Study, the gas chromatography/mass spectrometry, HPLC have been used for the determination of this pesticide in various samples [23].

Similarly, MCPA-2-ethylhexyl is skin and eye irritant. It is also highly toxic, hazardous and harmful even at very low concentration and effects the animals and environment both. Bromoxynil + MCPA are used to control post-emergence yearly broad-leaved herbs. Oftenly applied in mixture with other weedicides to enhance the control spectrum. The formulation types of Bromoxynil + MCPA are in EC (Emulsifiable Concentrate), SL (Soluble Liquid), SC (Suspension Concentrate), OD (Oil Dispersant) and SP (Soluble Powder). The various products of Bromoxynil + MCPA are found in brands which are being marketed by the name of 'Bronate' (Bromoxynil octanoate) (Bayer Crop Science), ‘MCPA Ester' (Dow Agro Sciences). While studying the environmental fate of MCPA it is found that in case of rats, following oral intake, MCPA fastly excreted and absorbed almost entirely in urine with only a small amount in faeces.

Only few techniques and methods have been found for determination of Bromoxynil and MCPA residues in urine, and canine plasma [24–27]. Determination in food and serum [28–31], In fruits and water was determined by mass-spectrometry [32–34]. Various methods of analysis have been reported in literature for bio degradation and determination in wheat samples [35–37].

Though only few methods have been reported previously for certain applications involving extraction and determination of Bromoxynil and MCPA residues in fruits, vegetables, wastewater, drinking water, human serum, municipal and industrial wastewater, rain water and river water but none of the RP-HPLC–UV method has still been developed which may be simple, economical for simultaneous determination of Bromoxynil and MCPA either in raw material and/or for dosages formulations.

Thus, the aim of present study was to develop an analytical method based on RP-HPLC–UV technique for simultaneous determination of Bromoxynil and MCPA both in pesticides pure, raw material and various dosage formulations. The basic purpose to develop this RP-HPLC–UV method is that most of the under-developed and developed countries have agrochemical industries. These industrial units have high performance liquid chromatography with UV detector (HPLC–UV) in their quality control laboratories which is commonly used instrument for determination of pesticides and is cheaper and easily handled as compared to florescent, MS and other equivalents in Liquid Chromatography. So, there is a need to develop a RP-HPLC–UV method which should be equally efficient, valid, precise and highly reproducible because not a single official method of analysis has still been reported for simultaneous determination of Bromoxynil and MCPA in CIPAC (Collaborative International Pesticide Analytical Council) [38], FAO (Food and agricultural Organization) [39] and in AOAC **(**Association of Official Agricultural Chemists**)** [40] at commercial/industrial scale.

**Fig. 1:**
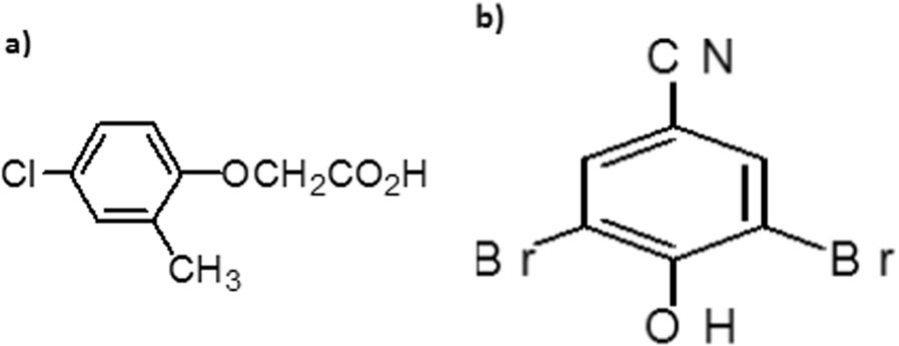
(**a**) Structure of MCPA (**b**) Structure of Bromoxynil

Figure 1a represents the structure of MCPA. The IUPAC name and Chemical name is (4-chloro-2-methylphenoxy) acetic acid and MCPA-2-ethylhexyl respectively while Fig. 1b represents the structure of Bromoxynil. Its IUPAC name and chemical name is 3, 5-dibromo-4-hydroxybenzonitrile; 3, 5-dibromo-4-hydroxyphenyl cyanide.

## Materials and method

### Chemicals and reagents

Highest purity analytical Reagent Grade chemicals were used during the whole experimental and practical work. Methanol HPLC gradient grade was purchased from Duksan Pure Chemicals Korea, Water HPLC Grade from VWR Chemicals (BDH) prolabo. Bromoxynil Octanoate analytical standard of known purity 99.4% (Equivalent to Bromoxynil = 68.31762%) was obtained from Chem. Services USA and that of MCPA-2-ethylhexyl 99.8% (Equivalent to MCPA = 64.00174%) was obtained from FLUKA Sigma Aldrich Germany. A well known mixture sample of 40% W/V EC (Emulsifiable Concentrate) Bromoxynil 20% (200 g/L) (from Bromoxynil Octanoate) and MCPA 20% (200 g/L) (from MCPA-2-Ethylhexyl) product marketed by the name of Cut Out, was collected from Solex Chemicals (Pvt) Ltd. Multan, Pakistan and some other formulations of EC (Emulsifiable Concentrate) and SC (Suspension Concentrate) of various concentrations of active ingredients of Bromoxynil and MCPA of different companies was collected from local market of Multan Pakistan.

### Instruments and apparatus

A filtration assembly (Glasco) with filtration pump was used for mobile phase filtration. Filter papers of 0.25 and 0.45 µm (Sartorius) were used for filtration of mobile phase. 42 No. filter paper was purchased from Sartorius used for filtration of sample. An ultrasonic water bath (GT Sonic model D3, China) was used for the extraction of the sample and standard analytes. Certified glassware was purchased from Iwaki Pyrex England were used during the whole analytical work. HPLC analysis of Bromoxynil + MCPA was performed with Shimadzu Japan HPLC system comprising of LC-20 AT pump with SPD-20A Ultra Violet-Visible detector. A zorbax 250 × 4.6 mm (i.d) packed C_18_ column with 5 µm (particle size) from Agilent Technology was set at normal room temperature. Isocratic elution was performed for the separation of Bromoxynil and MCPA contents by using the mobile phase (Methanol 90% + Water 10%). The optimized flow rate used during the analysis was 1.5 mL/min. Analyte volume injected was 20 µL. The micro glass syringe of 50 µl with stainless steel piston was purchased from SGE. The λ_max_ used for simultaneous detection of Bromoxynil and MCPA was 230 nm. Content %age of the Bromoxynil and MCPA analytes was detected by comparing retention time of analyte peak with retention time of external analytical standard peak. The Identification of Bromoxynil and MCPA analytes were by comparison of retention times of 5.798 min in case of Bromoxynil and 6.797 min in case of MCPA respectively.

### Preparation of calibration standard solutions of various concentrations for linearity curve

Stock solution of Bromoxynil and MCPA mixture 1000 mg/L from pure Bromoxynil 68.32% (From Bromoxynil Octanoate 99.4%) and MCPA 64.00% (from MCPA -2- Ethylhexyl 99.8%) from analytical standard of high purity was prepared with the trueness of ± 0.0001 mg/L into a separate 100 mL volumetric flask by taking weights of 146.4 and 156.3 mg of Bromoxynil and MCPA analytical standard respectively. The analytical standard of Bromoxynil + MCPA was dissolved into the 30 mL of mobile phase (Methanol 90% + Water 10%) by sonication moderately and then made up to the volume with mobile phase. Working standards of 150, 200, 250, 300 and 350 mg/L of both the Bromoxynil and MCPA in a 50 mL volumetric flask were prepared for linearity curve from the stock solution of 1000 mg/L of analytical standard solution by diluting with mobile phase (Methanol 90% + Water 10%) up to the 50 mL mark. All the working standard solutions were filtered with nylon membrane filter paper of 0.45 µm and analyzed on HPLC. The data was recorded in the form of the chromatograms. Three readings of each concentration point were taken and mean of each concentration point was used to draw the linearity curve.

### Preparation of standard solution

The 200 mg/L of Bromoxynil and MCPA standard sample solution mixture was prepared in 100 mL volumetric flask by taking the weights of 0.029 gm in case of Bromoxynil 68.30% (from Bromoxynil Octanoate 99.4%) and 0.032 gm in case of MCPA 64.00% (from MCPA-2-Ethylhexyl 99.8%) from pure analytical standard. Add 30 mL of mobile phase (Methanol 90% + Water 10%) first and sonicate it to dissolve completely. The solutions were diluted up to 100 mL with mobile phase (Methanol 90% + Water 10%) and shaken vigorously to make homogeneous solutions. The sample solution of Bromoxynil and MCPA were filtered through nylon membrane filter paper of 0.45 µm [41].

### Preparation of the sample solution

The 200 mg/L of Bromoxynil and MCPA pure contents (From CUTOUT 40% = Bromoxynil 20% (200 g/L) and MCPA 20% (200 g/L) EC W/V) from product sample was prepared by taking weight 0.1002 gm and diluting with mobile phase (Methanol 90% + Water 10%) in 100 mL volumetric flask. The product sample solution was manually shaken for one minute to attain homogeneity. The sample was filtered with membrane filter paper of 0.45 µm and maintained at lab room temperature (25–28 ℃) for analysis on HPLC and the data was recorded in the form of chromatograms. The percentage recovery was calculated by repeating the whole procedure thrice [41].

### HPLC conditions and method optimization

Different Chromatographic parameters were set by changing the various mobile phase compositions and rate of flow. By varying the ratios of HPLC gradient grade solvents for example methanol and water were set for the mobile phase optimization to obtain best separation of the analyte with good resolution. The flow rate of the mobile phase was changed between 0.5 and 1 mL/min at changing interval of 0.1 mL/min. During the whole analysis process, isocratic elution of mobile phase was followed. Degassing of mobile phase was done by ultrasonic water bath after passing through 0.45 µm nylon membrane filter paper using vacuum pump filtration system. The process of the separation of analyte was done by using C-18 column at the room temperature. Various wavelengths of UV range between 200 and 300 nm at the interval of 10 nm were tested to decide λ_max_ and optimum chromatographic responses to minimize interferences received from inert materials available in the formulated products. The optimum flow rate and mobile phase ratios were changed deliberately to perform the robustness test. Comparison of the results achieved by changing each parameter [42].

### Proposed method

RP-HPLC–UV system used was from Shimadzu Japan LC-20AT with SPD-20A detector where detector wavelength used was 230 nm and Column C18 Zorbax Agilent Technologies serial number 560562 (250 × 4.6 mm (i.d) × 5 µm). The mobile phase used was** (**Methanol 90% + Water 10%). The flow rate was maintained at 1.5 mL/min and the approximate retention time was observed to be 5.79 min for Bromoxynil and 6.797 min for MCPA pure active contents [42].

The Bromoxynil + MCPA contents were quantitatively determined by the use of pure external analytical standards of Bromoxynil and MCPA purchased from Chem Services USA and FLUKA Sigma Aldrich Germany respectively and by use of correction factor using the following Eq. 1 [41].

1 $$ {\text{Bromoxynil Octanoate contents \% }}\left( {\frac{{\text{w}}}{{\text{w}}}} \right){\text{X}}1 = { }{\raise0.7ex\hbox{${{\text{A}}_{2} {\text{x m}}_{1} {\text{ x P}}}$} \!\mathord{\left/ {\vphantom {{{\text{A}}_{2} {\text{x m}}_{1} {\text{ x P}}} {{\text{A}}_{{1{ }}} {\text{x m}}_{2} }}}\right.\kern-0pt} \!\lower0.7ex\hbox{${{\text{A}}_{{1{ }}} {\text{x m}}_{2} }$}} $$

Bromoxynil Octanoate Contents % (w/w) × 0.6873 (Factor to convert Bromoxynil Octanoate to Bromoxynil).

Bromoxynil Contents % (w/v) = Bromoxynil% (w/w) x Density of Bromoxynil Liquid sample of mixture (CUTOUT 40% W/V).

Where:

A_1_ = Average peak area of the Bromoxynil in the standard solution.

A_2_ = Average peak area of the Bromoxynil in the sample solution.

m_1_ = mass of Bromoxynil standard (mg).

m_2_ = mass of Bromoxynil sample (mg).

P = Purity of Bromoxynil analytical standard.

Similarly, the contents of MCPA% (w/v) can also be calculated from above equation used for calculating Bromoxynil active ingredient contents% (w/v).

While the factor used for the conversion of MCPA -2- Ethylhexyl to MCPA is 0.6413.

## Results

### Method validation

**Fig. 2:**
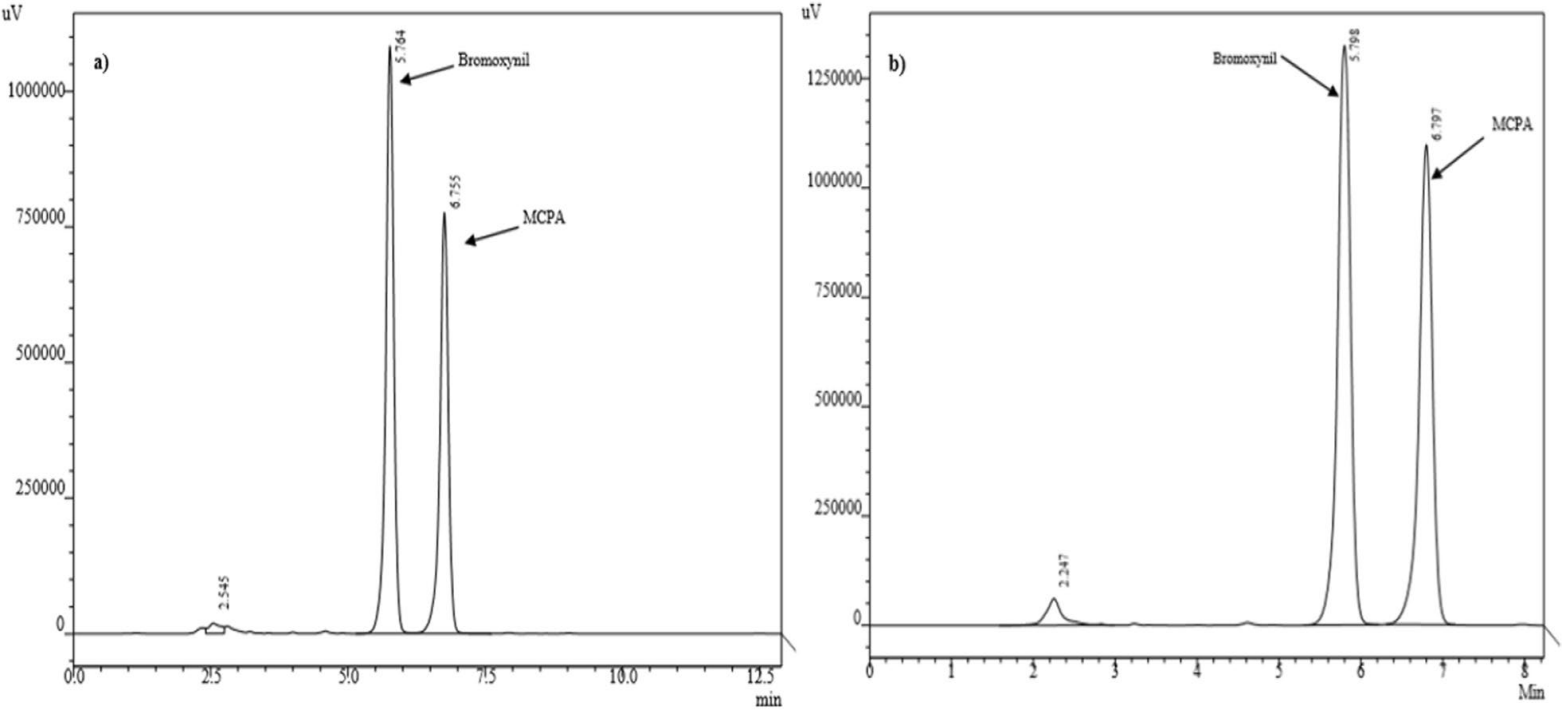
HPLC Chromatogram of the Bromoxynil and MCPA (**a**) Standard solution **b** Sample solution

The HPLC chromatograms of Bromoxynil and MCPA in Fig. 2a, b showed the same retention time (Bromoxynil = 5.7 min and MCPA 6.7 min) in analytical standard as well as in sample solution.

### Linearity for bromoxynil and MCPA

**Fig. 3:**
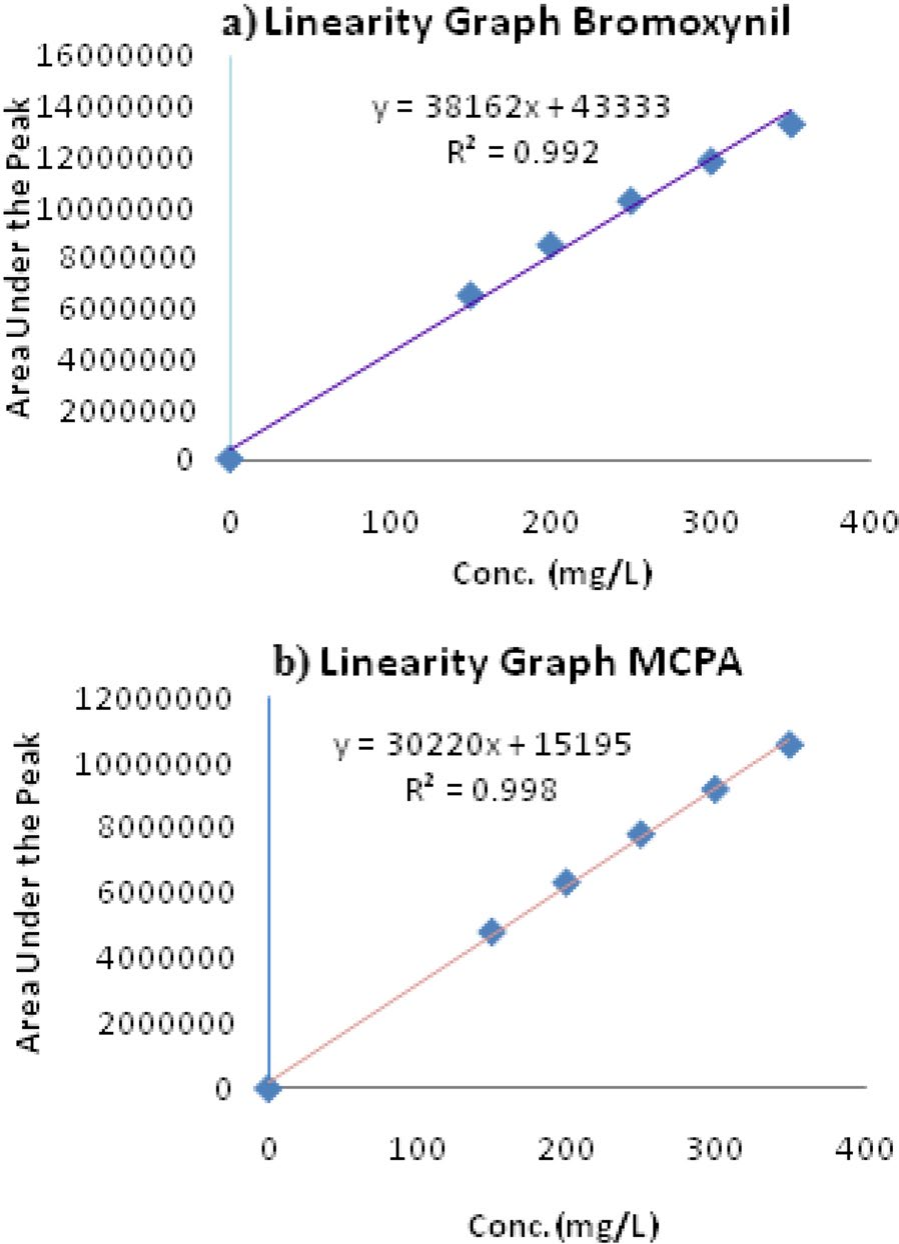
Linearity curves of the developed method for (**a**) Bromoxynil and (**b**) MCPA

Figure 3 (a and 3) describes the linearity curves for Bromoxynil and MCPA respectively which have been plotted between the concentration (mg/L) and peak area. The linearity of the method developed for the Bromoxynil and MCPA was evaluated by using various concentrations of 150, 200, 250, 300 and 350 mg/L of Bromoxynil and MCPA by taking three readings of each concentration point and mean of each concentration point used for calibration curve as shown in Table 1a, b. The value of correlation coefficient (R^2^) was 0.992 for Bromoxynil and 0.998 for MCPA. The R^2^ value shows that extraction has been verified by the HPLC method develop for analysis of Bromoxynil and MCPA simultaneously in pure Active Ingredient (A.I) in raw materials and pesticides dosage formulations.

**Table 1:** Linearity curve of the developed method for the Bromoxynil and MCPA

(a) Area under the peak of analytical standard solutions of various concentrations of bromoxynil for linearity curve		
Conc.(mg/L)	Peak area	Peak area mean
150	6504028	6490557
	6484190	
	6483453	
200	8486398	8481148.7
	8472233	
	8484815	
250	10229276	10236719.33
	10235442	
	10245440	
300	11794610	11811751
	11806984	
	11833659	
350	13293322	13282454.33
	13278442	
	13275599	

(b) Area under the peak of analytical standard solutions of various concentrations of MCPA for linearity curve		
Conc.(mg/L)	Peak area	Peak area mean
150	4822103	4806237.667
	4798486	
	4798124	
200	6333703	6328976.667
	6321892	
	6331335	
250	7801844	7811342.667
	7806942	
	7825242	
300	9168300	9187722.333
	9174948	
	9219919	
350	10570467	10551858.67
	10545896	
	10539213	

### Precision and specificity for bromoxynil and MCPA

For the assessment of the precision in method validation, five replicates of Bromoxynil and MCPA of 200 mg/L concentration were prepared. Table 2a the peak area for each analyte from HPLC data was calculated for measuring the value of standard deviation and relative standard deviation. Value of relative standard deviation from the measurement shows that it is within the permissible limit of (RSD =  ± 2%) as per NMT in accordance with ICH guidelines for method validation [43].

**Table 2:** Precision and Specificity of the developed method for the Bromoxynil and MCPA

(a) Precision of the developed method for the Bromoxynil and MCPA		
Standard Sample. #	Peak area of Standard Solution (Bromoxynil)	Peak area of Standard Solution (MCPA)
1	10886429	8370857
2	10892272	8375569
3	10902506	8394953
4	10891796	8375286
5	10889786	8373956
Average	10892558	8378124
Standard Deviation	6019	9592
RSD%	0.06%	0.11%

(b) Specificity of the developed method for the Bromoxynil and MCPA					
Products	Results in mixture	Mean result in soul sample		Recovery (80–120%)	Remarks
		Peak area of the standard solution	Peak area of the sample solution		
Bromoxynil	20.00%	10892558	14647428	99.53%^a^	Pass
		19.906%			
MCPA	20.00%	8378124.2	12429169	100.10%^a^	Pass
		20.02%			

^a^Average of five replicate

Table 2b shows that the developed method is specific for simultaneous determination of Bromoxynil and MCPA active ingredient contents which was monitored by use of blank solvent and analyte standard solution individually, in which peak was not observed and detected close to the peak of preferred analytes and other incipient and excipients. Hence, the method is proved to be highly specific.

### Trueness for bromoxynil and MCPA

The standard solutions of different concentrations were prepared to evaluate trueness of the developed method. The peak area was calculated like in case of linearity curve Fig. 3a for Bromoxynil. The values for slope and intercept for Bromoxynil were calculated. Similarly, the above said concentrations were prepared for MCPA standard solution and linearity curve was drawn as in Fig. 3b. Average of three replicate readings was calculated and results found by linearity curve. Table 3a, b shows the detail of areas under the peak for the standard and sample solutions. The percentage recovery data for Bromoxynil and MCPA as shown in Table 3a, b is within the standard acceptable limits (80–120%) which is the clear indication that the developed method is suitable for simultaneous determination of Bromoxynil and MCPA in the pesticides raw materials and in dosage formulations.

**Table 3:** Trueness of the developed method for the Bromoxynil and MCPA

(a) Trueness of the developed method for the Bromoxynil				
Conc. (mg/L)	Mean peak area of standard^a^	Mean peak area of sample^a^	Observed yield (mg/L)	Percentage recovery (%)
150	6490553	6461836	149.3	99.6%
200	8481149	8460868	199.5	99.8%
250	10236719	10202255	249.2	99.7%
300	11811751	11779311	299.2	99.7%
350	13282454	13231410	348.7	99.6%

(b) Trueness of the developed method for the MCPA				
Conc. (mg/L)	Mean peak area of standard^a^	Mean peak area of sample^a^	Observed yield (mg/L)	Percentage recovery (%)
150	4806238	4772312	149.0	99.3%
200	6328977	6288986	198.7	99.4%
250	7811343	7768557	248.6	99.5%
300	9187722	9125635	298.0	99.3%
350	10551859	10476843	347.5	99.3%

^a^Average of three replicate measurements

### Repeatability and reproducibility

In evaluating the Table 4a repeatability parameter for method validation of the Bromoxynil and MCPA it is indicated that by analyzing the Bromoxynil and MCPA analyte of both the standard and the sample solutions within different intervals of time by applying the same conditions on same instruments and analyst, the results showed the RSD% do not deviate the standard value of relative standard deviation (RSD% ≤ 2% as per NMT requirement for method validation). So, the proposed analysis method is found to be applicable by its repeatability.

**Table 4:** Repeatability and Reproducibility of the developed method for the Bromoxynil and MCPA

(a) Repeatability of the developed method for the Bromoxynil and MCPA					
Sr. #	Observations	Peak area (Bromoxynil)		Peak area (MCPA)	
		Solution of standard	Solution of sample	Solution of standard	Solution of sample
1	Reading 1	10886429	14648612	8370857	12455385
2	Reading 2	10892272	14660667	8375569	12380559
3	Reading 3	10902506	14661086	8394953	12381573
4	Reading 4	10891796	14651265	8375286	12469138
5	Reading 5	10889786	14615510	8373956	12459192
6	Average	10892558	14647428	8378124	12429169
7	Standard Deviation	6019	18687	9592	44200
8	RSD%	0.06%	0.13%	0.11%	0.36%

(b) Reproducibility of the developed method for the Bromoxynil and MCPA					
Sr. #	Observations	HPLC—20AT		HPLC—10AT	
		Bromoxynil	MCPA	Bromoxynil	MCPA
1	Reading 1	14648612	12455385	9263118	7598232
2	Reading 2	14660667	12380559	9213510	7560822
3	Reading 3	14661086	12381573	9223018	7560822
4	Reading 4	14651265	12469138	9227850	7573807
5	Reading 5	14615510	12459192	9238788	7576635
6	Average	14647428	12429169	9233257	7574064
7	Standard Deviation	18687	44200	19015	15342
8	RSD %	0.13%	0.36%	0.21%	0.20%

The reproducibility parameter for the developed HPLC method was performed on two HPLC units namely HPLC-20AT and HPLC-10AT from Shimadzu Corporation Japan. The Table 4b for Bromoxynil and MCPA shows the data obtained from Bromoxynil and MCPA contents from both HPLC units showed the relative standard deviation values at HPLC-20AT and HPLC-10AT. This clearly indicates that the developed method for the simultaneous determination for Bromoxynil and MCPA analyte did not deviate from the standard value of RSD ≤ 2%. So, the developed analytical method is found reproducible and fit for analyzing Bromoxynil and MCPA contents in both raw material and pesticide dosage formulations.

Table 5a, b clearly indicates that on analyzing the Bromoxynil + MCPA in various labs the results are specific, reproducible and repeatable with RSD values found within the international permissible and declared limits of RSD ≤ 2%.

**Table 5:** Reproducibility of the developed method for the Bromoxynil and MCPA with respect to various dosages formulations

(a) Reproducibility with respect to various dosages formulations for the Bromoxynil			
Formulation	Company	Proposed method	
		Recovery % age	RSD %
CUT OUT 40% (EC)*	A	99.50	0.18
Bromoxynil-MCPA	B	99.97	0.14
Bromoxynil-MCPA	C	100.04	0.16
Bromoxynil-MCPA	D	100.26	0.16
Bromoxynil-MCPA	E	100.09	0.24

(b) Reproducibility with respect to various dosages formulations for the MCPA			
Formulation	Company	Proposed Method	
		Recovery % age	RSD %
CUT OUT 40% (EC)^a^	A	99.88	0.15
Bromoxynil-MCPA (EC)^a^	B	100.05	0.21
Bromoxynil-MCPA (EC)^a^	C	100.10	0.22
Bromoxynil-MCPA (EC)^a^	D	100.35	0.17
Bromoxynil-MCPA (EC)^a^	E	100.02	0.15
Walter Super (SC)^a^	F	100.77	0.08

^a^Where EC stands for Emulsifiable Concentrate and SC stands for Suspension Concentrate

### Limit of detection and quantitation

The LOD and LOQ values for method validation of Bromoxynil and MCPA are shown in Table 6. Five replicate readings were taken of the standard solution (250 mg/L) both for the Bromoxynil and MCPA contents. The LOD and LOQ were obtained through signal to noise ratio 3:1 and 10:1 respectively [44].

**Table 6:** LOD and LOQ of developed analysis method for the Bromoxynil and MCPA

Readings	Bromoxynil (mg/L)	MCPA (mg/L)
1	266.200	252.610
2	267.0150	253.1800
3	266.7500	253.0100
4	266.4200	252.8000
5	267.040	253.210
Average	266.69	252.96
Standard Deviation	0.3690	0.2557
Śo = SQR(2)* so	0.52	0.36
LOD = 3* Śo	1.57	1.08
LOQ = 10* Śo	5.22	3.62

### Robustness

While performing robustness of the analytical method developed for the simultaneous determination of Bromoxynil and MCPA as shown in Table 7 (a and b), it was observed that by increasing flow rate of mobile phase from 1.5 to 1.7 mL/min the peak areas decreased, however, the RSD% remained within the standard prescribed limits (RSD ≤ 2%). While decreasing the flow rate of mobile phase from 1.5 to 1.3 mL/min the peak area increased. In this case, again the RSD% remained within the limit and did not cross the standard value (RSD ≤ 2%). Similarly, robustness of the developed method had also been evaluated by varying the mobile phase concentrations from Methanol: Water = 90: 10 (v/v) to Methanol: Water = 85: 15 (v/v). It had been noted that the peak area increased while the RSD% value did not deviate from the acceptable standard limit (RSD ≤ 2%). Whereas during the decrease of water ratio in the mobile phase (Methanol: Water = 90: 10) to (Methanol: Water = 95: 05) the peak area began decreasing but again the RSD% value showed no deviation from the standard acceptable limit of relative standard deviation value (RSD ≤ 2%). So, the developed method for simultaneous determination of Bromoxynil and MCPA was found fit and applicable in raw material and pesticide dosage formulations.

**Table 7:** Robustness of the developed method at the change of flow rate and mobile phase for the Bromoxynil and MCPA

(a) Robustness of the developed method at the change of flow rate and mobile phase for the Bromoxynil						
Sample No	Change of Flow Rate			Change of Mobile Phase		
	Peak area at 1.3 mL/min	Peak area at 1.5 mL/min	Peak area at 1.7 mL/min	Methanol: Water 95: 05	Methanol: Water 90: 10	Methanol: Water 85: 15
01	16899261	14648612	13095952	11794631	14648612	19704492
02	16893790	14660667	13112501	11793598	14660667	19731947
03	16880153	14661086	13109821	11759541	14661086	19757886
04	16901669	14651265	13109991	11777437	14651265	19760224
05	16927612	14615510	13108926	11726960	14615510	19762280
Mean	16900497	14647428	13107438	11770433	14647428	19743366
Standard deviation	17299	18687	6557	28195	18687	24972
% RSD	0.10%	0.13%	0.05%	0.24%	0.13%	0.13%

(b) Robustness of the developed method at the change of flow rate and mobile phase for the MCPA						
Sample No	Change of Flow Rate			Change of Mobile Phase		
	Peak area at 1.3 mL/min	Peak area at 1.5 mL/min	Peak area at 1.7 mL/min	Methanol: Water 95: 05	Methanol: Water 90: 10	Methanol: Water 85: 15
01	14290800	12455385	11060939	9951291	12455385	16818457
02	14279376	12380559	11058719	9950908	12380559	16843060
03	14267751	12381573	11057852	9921681	12381573	16875223
04	14294707	12469138	11051754	9910312	12469138	16883146
05	14342259	12459192	11047533	9899583	12459192	16904524
Mean	14294979	12429169	11055359	9926755	12429169	16864882
Standard deviation	28451	44200	5544	23557	44200	34072
% RSD	0.20%	0.36%	0.05%	0.24%	0.36%	0.20%

### Summary of validation parameters for bromoxynil

#### Summary of validation parameters for MCPA

Summary of the validation parameters for bromoxynil and MCPA has been shown in Tables 8 and 9 representing the various validation parameters in tabulated form.

**Table 8:** Summary of Validation Parameters for Bromoxynil

Parameters		Results (Bromoxynil)		Acceptance limit
Linearity		Correlation Coefficient = 0.992		Correlation Coefficient NLT^a^ 0.97
Precision		0.06% RSD		% RSD NMT^b^ 2.0
Trueness		Conc. (mg/L)	% Recovered	% Recovery within 80–120%
		150	99.6%	
		200	99.8%	
		250	99.7%	
		300	99.7%	
		350	99.6%	
Repeatability	(with respect to Instrument and Analyst)	0.13% RSD		RSD ≤ 2.0%
Reproducibility	(with respect to Instrument)	HPLC–20AT	HPLC–10AT	
		0.21% RSD	0.13% RSD	
	[with respect to various Labs (ILC)]	Average 0.18%RSD		
Detection and quantitation limit		LOD	LOQ	−
		1.57 mg/L	5.22 mg/L	
Robustness		Change	% RSD	% RSD NMT 1.5
		Flow rate = 1.3 mL	0.10%	
		Flow rate = 1.5 mL	0.13%	
		Flow rate = 1.7 mL	0.05%	
		(Mobile Phase) Methanol: Water 950: 50	0.24%	
		(Mobile Phase) Methanol: Water 900: 100	0.13%	
		(Mobile Phase) Methanol: Water 850: 150	0.13%	

^a^Not Less than in accordance to the ICH Analytical procedures developments Guidelines [43]

^b^Not More than in accordance to the ICH Analytical procedures developments Guidelines [43]

**Table 9:** Summary of Validation Parameters for MCPA

Parameters		Results (MCPA)		Acceptance limit
Linearity		Correlation Coefficient = 0.998		Correlation Coefficient NLT 0.97
Precision		0.11% RSD		% RSD NMT 2.0
Trueness		Conc. (mg/L)	% Recovered	% Recovery within 80–120%
		150	99.3%	
		200	99.4%	
		250	99.5%	
		300	99.3%	
		350	99.3%	
Repeatability	(with respect to Instrument and Analyst)	0.36% RSD		
Reproducibility	(with respect to Instrument)	HPLC–20AT	HPLC–10AT	RSD ≤ 2.0%
		0.36% RSD	0.20% RSD	
	[with respect to various Labs (ILC)]	Average	0.16% RSD	
Detection and quantitation limit		LOD	LOQ	–
		1.08 mg/L	3.62 mg/L	
Robustness		Change	% RSD	% RSD NMT 1.5
		Flow rate = 1.3 mL	0.20%	
		Flow rate = 1.5 mL	0.36%	
		Flow rate = 1.7 mL	0.05%	
		(Mobile Phase) Methanol: Water 950: 50	0.24%	
		(Mobile Phase) Methanol: Water 900: 100	0.36%	
		(Mobile Phase) Methanol: Water 850: 150	0.20%	

## Discussion

In the field of analytical research, the method development is an extremely important area of the study. In industrial research, especially in pesticides and in pharmaceutical there is always a requirement of method development for the different analytes through the well known chromatographic and spectroscopic techniques. The developed method should be unique, novel, easy, cheaper, efficient, reproducible and valid to the particular analyte. In the present study, very simple, novel, unique, cheaper, efficient and reproducible HPLC analysis method has been developed for the simultaneous determination and quantification of the Bromoxynil and MCPA contents both in raw material and dosage formulations. Analytical standard solution of both the Bromoxynil and MCPA were analyzed too. It was found that mobile phase consisting of Methanol: Water = 90: 10 (v/v) is best solubilizing media [45]. It was also noted from the chromatograms that the retention time is the same for analytical standard and sample solutions in the both analyte peaks of Bromoxynil and MCPA.

The optimization of parameters was done first and then validation of the method completed in terms of suitability of the system, linearity, trueness, precision, repeatability, reproducibility, LOD, LOQ and robustness. In an analytical method validation, linearity is considered as the first step [46]. The value of the precision was found in the acceptable limit and was considered as best than reported in the previous analysis methods for these analytes.

In the method validation, the parameter of trueness was also studied. For each concentration of Bromoxynil, the percentage recovery was calculated by comparing the peak area of standard solution to that of the sample solution. The trueness were determined in terms of recovery percentage 99.70% ± 0.084 (n = 5) at various concentrations and for MCPA 99.40% ± 0.09 (n = 5) as described in Table 2. Hence, the proposed method is accurate with excellent recoveries for both Bromoxynil and MCPA at different concentrations [47]. So, under the optimized conditions, the developed method indicates that the simultaneous determination of the Bromoxynil and MCPA was accurate and reproducible with excellent recoveries of samples from various sources.

The repeatability of proposed method was also carried out on the same instruments with same analyst and instrumental conditions with excellent outcome of relative standard deviation (RSD%) of 0.06 and 0.13% for standard and sample solution respectively for Bromoxynil while for MCPA, RSD is 0.11% and 0.36% for standard and sample solutions respectively [48]. Hence, these values of RSD% were found within the standard acceptable limits (RSD ≤ 2%) and showed no deviation. So, the developed method is repeatable and fit to apply both in the raw material and pesticide dosage formulations.

The reproducibility of the proposed method was also carried out at HPLC 10-AT VP and HPLC 20-AT with SPD 10A and 20A UV–Visible detector respectively and excellent results was found in terms of relative standard deviation at HPLC-20AT (Bromoxynil: RSD = 0.13% and MCPA: RSD = 0.36%) and at HPLC-10AT (Bromoxynil: RSD = 0.21% and MCPA: RSD = 0.20%) which clearly indicated that the developed method for simultaneous determination for Bromoxynil and MCPA did not deviate the acceptable limit of RSD ≤ 2%. So, the developed analytical method is found fit and reproducible for simultaneously determination of Bromoxynil and MCPA in both the raw material and pesticide dosage formulations. The value of LOD and LOQ was found for Bromoxynil (LOD = 1.57 mg/L and LOQ = 5.22 mg/L) and for MCPA (LOD = 1.08 mg/L and LOQ = 3.62 mg/L) of the developed method [49].

While performing robustness of the developed method, it was observed that by increasing the flow rate of mobile phase from 1.5 to 1.7 mL/min the peak area decreased. While the RSD% remained within the standard prescribed limits (RSD ≤ 2%) [50]. While lowering the flow rate of mobile phase from 1.5 to 1.3 mL/min, the peak area increased. In this case again the RSD% remained within the limit and did not cross the standard value (RSD ≤ 2%). This can be due to the fact that the analytes pass through the system very rapidly without much retention at higher flow rate which results in the smaller peak area but the relative standard deviation values (RSD%) remain still in standard acceptable limits (RSD ≤ 2%) even at the higher flow rate [51]. Similarly, robustness had also been evaluated by varying the mobile phase concentrations from (Methanol: Water = 90: 10) to (Methanol: Water = 85: 15) it had been noted that the peak area increased while the RSD% value did not deviate the acceptable standard limit (RSD ≤ 2%). Whereas by decreasing water ratio in the mobile phase (Methanol: Water = 90: 10) to (Methanol: Water = 95: 05) the peak areas decreased but again the RSD% showed no deviation from the acceptable limit (RSD ≤ 2%) [52]. So, the developed method for simultaneous determination of Bromoxynil and MCPA was found fit and applicable both in raw material and pesticide dosage formulations.

## Conclusions

The developed and validated reversed phase HPLC–UV method has been found robust and efficient for the simultaneous determination of Bromoxynil + MCPA mixture in raw materials and various dosage formulations in quality control laboratories. This chromatographic method follows analysis in isocratic elution mode. In comparison to the analytical methods earlier reported in the literature, the developed method is cheaper, very simple, accurate, repeatable and reproducible. The method validated according to ICH and Eurachem guidelines and showed reliable chromatographic characteristics. This method can be applied directly without any prior separation and pretreatment of samples with less retention time without interfering the desired analytes. So, the developed reversed phase HPL-UV method can widely be used to the real samples analysis at commercial scale in pesticide industry.

## Data Availability

The datasets used and/or analyses during the current study are available from the corresponding author on reasonable request.
